# Reduced fetal MAPSE and TAPSE in gestational diabetes mellitus: a prospective case–control study

**DOI:** 10.1007/s11845-026-04426-8

**Published:** 2026-04-29

**Authors:** Koray Gök, Merve Baştan, Rahime Tüten, Mustafa Doğan Özçil, Işın Erdoğan, Selçuk Özden, Abdullah Tüten

**Affiliations:** 1 Department of Obstetrics and Gynecology, Cerrahpasa Faculty of Medicine, Istanbul University-Cerrahpasa, Istanbul, Turkey; 2 Department of Obstetrics and Gynecology, Sakarya Research and Training Hospital, Sakarya, Turkey; 3 Department of Pediatric Cardiology, Bursa City Hospital, Bursa, Turkey; 4 Department of Obstetrics and Gynecology, Faculty of Medicine, Hatay Mustafa Kemal University, Antakya, Turkey; 5 Department of Obstetrics and Gynecology, Sanliurfa Education and Training Hospital, Sanliurfa, Turkey

**Keywords:** Annular plane systolic excursion, Fetal cardiac function, Fetus, Gestational diabetes mellitus, Ultrasonography

## Abstract

**Objective:**

To compare mitral and tricuspid annular plane systolic excursions (MAPSE and TAPSE, respectively) by using M-mode ultrasonography in fetuses of patients with diet-controlled GDM, insulin-treated GDM and a control group.

**Methods:**

This prospective case–control study enrolled 80 women with Gestational diabetes mellitus (GDM) and 95 healthy pregnant controls. Fetal MAPSE and TAPSE were obtained under standardized conditions by experienced operators using M-mode ultrasonography.

**Results:**

Fetal mitral annular plane systolic excursion (MAPSE) and tricuspid annular plane systolic excursion (TAPSE) values were found to be significantly lower in the GDM group compared with the control group (*p* < 0.001). In analyses evaluating GDM cases within themselves, fetal MAPSE and TAPSE values were found to be more significantly reduced in the insulin-treated GDM group compared to the diet-controlled GDM group.

**Conclusion:**

Fetal MAPSE and TAPSE values, measured by M-mode ultrasonography, were found to be significantly lower in the GDM group compared to the control group. The more pronounced decrease in these values, particularly in insulin-treated GDM cases, suggests that M-mode-derived MAPSE and TAPSE may serve as practical adjunctive markers of altered fetal cardiac function in GDM; however, their routine clinical use requires further validation with reproducibility analyses and postnatal outcome data.

## Introduction

Gestational diabetes mellitus (GDM) is defined as a glucose metabolism disorder that first appears in the second or third trimester of pregnancy in women without pre-existing diabetes diagnosed before pregnancy, and usually returns to normal in the postpartum period [1]. The prevalence of GDM shows a wide distribution among centers depending on the diagnostic criteria used, screening strategies, and the characteristics of the population in which the study is conducted, and is reported in the literature to be between 2 and 38% [2]. Due to its association with an increased risk of morbidity for the mother, fetus, and newborn, achieving metabolic control during pregnancy in pregnant women diagnosed with GDM is of clinically critical importance.

In diabetic pregnancies, maternal hyperglycemia is considered the main pathophysiological factor in the development of embryopathy and fetopathy. Hyperglycemia, especially starting in the periconception period and continuing throughout the first trimester, has been shown to be strongly associated with major congenital anomalies affecting multiple organ systems, primarily the cardiac structure and neural tube [3]. In contrast, gestational diabetes mellitus, which develops in the later stages of pregnancy, is reported to be associated more with fetal overgrowth and hypertrophic cardiomyopathy, which is part of the diabetic fetopathy spectrum [3]. It is suggested that maternal hyperglycemia chronically stimulates the beta cells of the fetal pancreas, leading to fetal hyperinsulinemia; as a result, fetal basal metabolic rate increases, oxygen consumption rises, and relative intrauterine hypoxia can develop [3]. Fetal cardiac structure and function are one of the main systems affected by the diabetic intrauterine environment due to their sensitivity to hyperinsulinemia and hypoxia. Although myocardial hypertrophy is a frequently reported finding in fetuses and newborns of diabetic mothers, it has been shown that subclinical impairments in fetal heart function may be present even in cases where no significant increase in myocardial thickness is detected and metabolic control is adequate throughout pregnancy [3, 4]. These findings demonstrate that in diabetic pregnancies, morphological evaluation alone is insufficient; fetal cardiac function should also be assessed using sensitive and quantitative parameters [4].

Conventional and advanced ultrasonographic techniques have been described for the assessment of fetal cardiac function. Mitral and tricuspid annular plane systolic excursion (MAPSE and TAPSE), measured by conventional M-mode ultrasonography, are considered parameters that reflect the longitudinal systolic function of the fetal heart, have high reproducibility, and are clinically applicable [5]. Studies evaluating the clinical benefit of fetal MAPSE and TAPSE measurements in various diseases, including pregestational and gestational diabetes, during the antenatal period are available in the literature [6–16]. However, it is noteworthy that studies conducted in homogeneous patient groups, including only pregnant women diagnosed with gestational diabetes mellitus and free from the significant effects of pregestational diabetes on early embryonic development, cardiac morphogenesis, and placental function, are limited.

The aim of this study was to evaluate the effectiveness of fetal MAPSE and TAPSE measurements in pregnancies diagnosed solely with gestational diabetes mellitus, independently of the potential confounding effects of pregestational diabetes, and also to reveal possible differences in these parameters among clinical subgroups of pregnant women with GDM.

## Materials and methods

This prospective case–control study included pregnant women diagnosed with gestational diabetes mellitus (GDM) who were admitted to the Perinatology Unit of the Department of Obstetrics and Gynecology at Sakarya University Training and Research Hospital between February 2021- February 2022 and who delivered at the same institution (n = 80). The control group consisted of healthy pregnant women at comparable gestational ages with no obstetric or medical complications (n = 95). Clinical and demographic data for both groups were obtained from hospital medical records. The study was approved by the Local Ethics Committee of Sakarya University Faculty of Medicine (approval no.: E16214662-050.01.04–6818-17,date: 5 February 2021).

Pregnant women with preeclampsia, pregestational diabetes mellitus, or any known chronic systemic disease—including chronic hypertension, chronic liver disease, chronic kidney disease, or rheumatologic disorders—were excluded. Additional exclusion criteria comprised HIV infection or other chronic inflammatory diseases, amniotic fluid abnormalities (polyhydramnios or oligohydramnios), preterm labor, premature rupture of membranes (PPROM), suspected fetal macrosomia based on ultrasonographic assessment, fetal structural or chromosomal anomalies, antenatal corticosteroid exposure, Doppler ultrasound findings suggestive of placental insufficiency, and an estimated fetal weight below the 10th percentile on sonographic evaluation.

The diagnosis of gestational diabetes mellitus was established between 24 and 28 weeks of gestation using a 75-g oral glucose tolerance test (OGTT). GDM was defined when at least one plasma glucose value exceeded the diagnostic thresholds (fasting ≥ 92 mg/dL, 1-h ≥ 180 mg/dL, or 2-h ≥ 153 mg/dL) [1].

All ultrasonographic examinations were conducted by experienced sonographers (KG, MB) using standardized equipment (Voluson 730 and Voluson E6; GE Medical Systems, Milwaukee, WI, USA) to ensure measurement consistency and to minimize operator-related bias. All measurements were performed in the absence of fetal movements. A four-chamber view was acquired with clear visualization of both atrioventricular annuli, and the M-mode cursor was aligned as parallel as possible to the longitudinal motion of the ventricular free wall. TAPSE was defined as the maximal systolic displacement of the lateral tricuspid annulus toward the cardiac apex, and MAPSE as the maximal systolic displacement of the lateral mitral annulus toward the cardiac apex. Care was taken to avoid foreshortening and oblique insonation, and the same standardized acquisition approach was used in all cases. Fetal mitral annular plane systolic excursion (MAPSE) and tricuspid annular plane systolic excursion (TAPSE) measurements were obtained in accordance with previously published and validated protocols [5, 6].

Statistical analyses were carried out using SPSS software version 24.0 (SPSS Inc., Chicago, IL, USA). Data distribution was assessed using the Kolmogorov–Smirnov test. Variables showing normal distribution were presented as mean ± standard deviation, whereas non-normally distributed variables were expressed as median and interquartile range (IQR). Comparisons between two independent groups were performed using the independent samples t-test for parametric data and the Mann–Whitney U test for non-parametric data. Comparisons among multiple groups were conducted using the Kruskal–Wallis test followed by Bonferroni-adjusted post hoc analysis. A Bonferroni-corrected alpha level < 0.05 and a p-value < 0.05 for other analyses were considered statistically significant.

## Results

Baseline characteristics of the gestational diabetes mellitus (GDM) group and the control group, as well as comparisons of fetal MAPSE and TAPSE values between the two groups, are presented in Table 1. There were no statistically significant differences between the GDM and control groups with respect to maternal age, body mass index (BMI), or gestational age at the time of ultrasonographic assessment (all *p* > 0.05). Fetal mitral annular plane systolic excursion (MAPSE) and tricuspid annular plane systolic excursion (TAPSE) values measured using M-mode ultrasonography were found to be significantly lower in the GDM group compared with the control group **(both *p* < 0.001)**.

**Table 1:** Comparison of baseline characteristics and fetal MAPSE and TAPSE values between the gestational diabetes mellitus group and the control group

Variables	Gestational diabetes group (n:80)	Control group (n:95)	p-value
Maternal age (years)	31 (20–41)	29 (19–42)	0.082
Body mass index (BMI) (kg/m^2^)	26.85 (23.5–35.1)	26.4 (22.4–36.8)	0.119
Gestational age at the time of the study (weeks)	30.56 (29.14–32)	30.56 (29.28–32)	0.207
MAPSE (mm)	4.1 (3.8–5.3)	5.5 (4.7–5.8)	** < 0.001**
TAPSE (mm)	5.85 (5.4–6.4)	6.8 (6–7.5)	** < 0.001**

Data are expressed as median (minimum–maximum). BMI, body mass index; MAPSE, mitral annular plane systolic excursion; TAPSE, tricuspid annular plane systolic excursion

Bold p-values indicate statistical significance

Comparisons of fetal MAPSE and TAPSE values among diet-controlled GDM, insulin-treated GDM, and control groups were performed using the Kruskal–Wallis test, revealing statistically significant differences among the three groups **(both *p* < 0.001)** (Table 2). Subsequent pairwise comparisons using the Mann–Whitney U test demonstrated that fetal MAPSE and TAPSE values differed significantly between all three groups **(all *p* < 0.001)**.

**Table 2:** Comparison of fetal MAPSE and TAPSE among the control group, diet-controlled gestational diabetes group, and insulin-treated gestational diabetes group

Variables	Control group (n:95)	Diet-controlled gestational diabetes (n:40)	Insulin-treated gestational diabetes (n:40)	p-value
MAPSE (mm)	5.5 (4.7–5.8)	4.6 (4–5.3)	4 (3.8–4.3)	** < 0.001**
TAPSE (mm)	6.8 (6–7.5)	6 (5.8–6.4)	5.6 (5.4–5.9)	** < 0.001**

Data are expressed as median (minimum–maximum).: MAPSE, mitral annular plane systolic excursion; TAPSE, tricuspid annular plane systolic excursion

Bold p-values indicate statistical significance

In the gestational diabetes mellitus (GDM) group, HbA1c levels demonstrated a statistically significant moderate negative correlation with both fetal mitral annular plane systolic excursion (MAPSE) and tricuspid annular plane systolic excursion (TAPSE) (*r* = − 0.433, *p* < 0.001 and *r* = − 0.452, *p* < 0.001, respectively) (Table 3).

**Table 3:** Correlation between HbA1c and fetal MAPSE and TAPSE in the GDM group

Variables	Correlation coefficient (r)	p-value
HbA1c—Fetal MAPSE	−0.433	** < 0.001**
HbA1c—Fetal TAPSE	−0.452	** < 0.001**

HbA1c, glycated hemoglobin; MAPSE, mitral annular plane systolic excursion; TAPSE, tricuspid annular plane systolic excursion. Bold p-values indicate statistical significance

## Discussion

This prospective case–control study demonstrated that fetal mitral annular plane systolic excursion (MAPSE) and tricuspid annular plane systolic excursion (TAPSE) values were statistically significantly lower in pregnancies diagnosed with gestational diabetes mellitus (GDM) compared to the control group. Furthermore, in analyses evaluating GDM cases within themselves, fetal MAPSE and TAPSE values were found to be more significantly reduced in the insulin-treated GDM group compared to the diet-regulated GDM group. Together, these findings support the concept that longitudinal ventricular systolic function may already be altered in fetuses exposed to the diabetic intrauterine environment, even in the absence of major structural anomalies or overt placental insufficiency.

Assessment of fetal cardiac function is increasingly used in fetal monitoring and assessment of fetal well-being. Longitudinal cardiac motion is determined by longitudinal myocardial fibers, which are most sensitive to conditions such as hypoxia and pressure/volume overload, and is considered a very sensitive and early indicator of cardiac dysfunction [10, 17, 18]. Longitudinal motion of the fetal heart can be assessed using various techniques, including M-mode ultrasound. M-mode ultrasound, a simple, sensitive, and reproducible method, can measure maximum mitral and tricuspid annular plane systolic excursions (MAPSE and TAPSE, respectively), and these parameters can provide information about longitudinal cardiac motion [6, 18]. Gestational diabetes mellitus is known to lead to complex pathophysiological changes, and consequently, the fetal heart is affected. In the fetal heart, since longitudinal myocardial fibers are the first to be affected by these changes, we used these parameters as practical markers of fetal longitudinal systolic performance in GDM pregnancies.

The performance of fetal MAPSE and TAPSE values ​​in demonstrating fetal cardiac function has been investigated in various complicated pregnancies. In a study conducted on fetuses with intrauterine growth restriction (IUGR), fetal MAPSE and TAPSE values ​​were reported to be significantly reduced compared to the control group, and it was stated that these values ​​may be sensitive in detecting cardiac dysfunction due to placental insufficiency [6]. In two separate studies conducted on monochorionic twins complicated by twin-twin transfusion syndrome, it was found that MAPSE and TAPSE values were reduced in both the recipient and the donor, indicating fetal cardiac dysfunction [7, 8]. In one study, fetal MAPSE and TAPSE Z-scores were found to be lower in fetuses with heart failure compared to normal fetuses, and it was stated that these scores can be used as a marker in both evaluating the systolic function of the heart and determining the severity of the failure [9]. In a recent study by Hendem et al. investigating fetal cardiac function in preeclamptic pregnancies, 48 healthy pregnant women between 32–34 weeks were compared with 48 preeclamptic pregnant women. In this study, fetal MAPSE and TAPSE values were reported as decreased in the preeclamptic group compared to the control group [10]. These data support the biological plausibility of our findings and suggest that impaired longitudinal systolic motion may represent a common adaptive response of the fetal myocardium to different adverse intrauterine conditions.

Looking at the literature, there are studies that investigate fetal MAPSE and TAPSE values together in pregnancies complicated by diabetes (pregestational and gestational), another high-risk form of pregnancy. In a study that included only patients with pregestational diabetes (17 type 1 DM, 51 type 2 DM), fetal MAPSE and TAPSE values were found to be statistically significantly lower in cases of poor glycemic control, similar to our study. This study was conducted at 30–32 weeks of gestation, similar to our study, and the researchers stated that these values may be useful in evaluating the systolic functions of the fetal heart [11]. In a study conducted between pregestational diabetics and healthy pregnant women, fetal MAPSE and TAPSE values were found to be statistically significantly lower in the pregestational diabetes group. However, unlike our study, this study included a total of 90 patients, 45 from each group, between 20 and 34.6 weeks of gestation. In this study, unlike ours, the researchers did not perform these measurements at similar gestational weeks between the two groups, nor did they compare well-controlled and poorly-controlled diabetes groups. However, they stated that fetal MAPSE and TAPSE measurements are a valuable tool in determining the risk of developing fetal early heart failure, especially in diabetic pregnant women, during the antenatal period and that they can be integrated into clinical practice [12]. In a study by Lee-Tannock et al., in which 50 diabetic pregnant women (34 GDM, 7 type 1 DM, 9 type 2 DM) were compared with 126 healthy pregnant women, fetal MAPSE and TAPSE values were measured every four weeks between 18 and 40 weeks of gestation. In this study, unlike our study, patients were evaluated over a wider gestational week, and it is noteworthy that there was a significant difference in BMIs between the groups. The researchers did not find different fetal MAPSE and TAPSE values between the groups and stated that this may be due to the fact that glycemic control was not well evaluated in their study [13]. In the study by Song et al., which compared 60 GDM patients with 60 healthy pregnant women and found a significant difference in BMI between the groups, although fetal MAPSE and TAPSE values were lower in the GDM group, no statistically significant difference was observed between the groups. The researchers stated that studies using larger sample sizes are needed to evaluate the value of the small differences observed [14]. In the study by Yovera et al., 43 GDM and 71 healthy pregnant women were evaluated between 24 + 0 and 32 + 0 weeks, and 69 GDM and 153 healthy pregnant women were evaluated between 32 + 1 and 40 + 1 weeks. It was stated that 27 patients in the GDM group were treated with insulin and/or metformin, while the rest had optimal blood sugar control with diet. Although a different agent (Metformin) was used in the treatment, unlike our study, this study found that fetal MAPSE and TAPSE values were significantly reduced in the GDM group compared to healthy pregnant women, similar to our study [15]. In another recent study, 87 patients were examined at 34–37 weeks of gestation, including 46 GDM patients (22 diet-regulated GDM, 24 insulin-regulated GDM) and 41 healthy pregnant women. Unlike our study, this study, in which a smaller number of patients were evaluated at a later gestational week, found no difference between the groups in terms of fetal MAPSE and TAPSE values [16]. Looking at these studies, it was observed that BMI was not specified in 2 of them [12, 16], there were differences between the groups in terms of BMI in 3 of them [11, 13, 14], and in the study by Yovera et al. [15], the evaluation was done after correcting for BMI. In our study, to avoid the confounding effect of obesity, the groups were formed from subjects with similar BMIs. In addition, in our study, GDM patients were divided into groups within themselves at a specific gestational week, namely those treated with insulin and those controlled by diet, and a larger number of patients were included in the study, unlike other studies. This allowed us to obtain our results in a more homogeneous population. However, we believe that the differing results between these studies may be due to variations in sample size, maternal body mass index (BMI), gestational age at which ultrasonographic evaluation was performed, level of glycemic control, and treatment methods used.

Although gestational diabetes mellitus is known to affect the fetal heart and cause a number of changes in its functions, the underlying pathophysiological mechanisms remain unclear. However, the major teratogen responsible for all these changes is hyperglycemia. Animal studies have shown that exposure to a hyperglycemic environment during the intrauterine period leads to changes in fetal myocardial remodeling and myocardial metabolism [19]. In addition, hyperglycemia, as a metabolic stressor, leads to endothelial dysfunction, reduces the angiogenic activity of endothelial cells, creates a hypoxic environment, and ultimately reduces the survival of endothelial cells [20]. In GDM, changes in lipid metabolism (increased fatty acid load), increased fetal cardiac load, and changes in placental anatomy and physiology (impaired villus development, placental malperfusion) also contribute to the disrupted intrauterine environment created by hyperglycemia. As a result of all these, insufficient oxygen and nutrients are supplied to the fetus, impairing myocardial contractility [3, 21, 22]. In light of all these findings, we believe that the decreased fetal MAPSE and TAPSE values, especially in insulin-treated GDM accompanied by a worse hyperglycemic environment, are a response to the impaired intrauterine environment in GDM. This is because the first structures affected in the fetal heart in the impaired intrauterine environment are the longitudinal myocardial fibers [23].

From a clinical perspective, our findings do not justify an immediate change in routine obstetric management on the basis of MAPSE or TAPSE alone. Rather, they suggest that these measurements may help identify a subgroup of fetuses exposed to GDM, particularly insulin-treated GDM, who may warrant closer functional cardiac assessment within a comprehensive fetal evaluation. Before these parameters can be incorporated into routine clinical practice, additional studies are needed to establish reproducibility, gestational age-specific reference thresholds in GDM, and their relationship with neonatal outcomes and postnatal echocardiographic findings.

This study has several strengths, including its prospective design, the comparable demographic characteristics of the groups, and the separate analysis of diet-controlled and insulin-treated GDM. However, the findings should be interpreted in light of important limitations. First, the exclusion of fetuses with estimated fetal weight below the 10th percentile, Doppler abnormalities suggestive of placental insufficiency, polyhydramnios, and suspected macrosomia resulted in a selected study population; therefore, the results may not be generalizable to all GDM pregnancies. Second, a formal a priori sample size calculation was not performed. Third, intraobserver and interobserver reproducibility analyses were not available, so reproducibility cannot be claimed on the basis of the present data. Fourth, postnatal echocardiographic follow-up and neonatal cardiac outcome data were not available. These points should be addressed in future studies.

In conclusion, fetal MAPSE and TAPSE values measured by M-mode ultrasonography were lower in the GDM group than in the control group, with the greatest reduction observed in insulin-treated GDM. These findings suggest that MAPSE and TAPSE may be useful adjunctive markers of altered fetal longitudinal systolic function in GDM. Nevertheless, their clinical utility should be interpreted cautiously until their reproducibility and prognostic value are confirmed in larger studies that include postnatal follow-up.

## Data Availability

The datasets generated and/or analyzed during the current study are used in the paper.
